# Impact of intravascular ultrasound guidance on radiation exposure during endovascular abdominal aortic aneurysm repair: Single-center experience

**DOI:** 10.1016/j.jvscit.2026.102548

**Published:** 2026-10-07

**Authors:** Mahetab Shehata, Ahmed Elshiekh, Asif Mahmood, Nicholas Matharu, Ahmed Hassan, Chris Koller, Ashraf Elsharkawy

**Affiliations:** a Department of Vascular Surgery, University Hospitals of Coventry and Warwickshire, Coventry, West Midlands, UK; b Department of Radiology Physics, University Hospitals of Coventry and Warwickshire, Coventry, West Midlands, UK

**Keywords:** Radiation safety, Endovascular repair of aortic aneurysm, Intravascular ultrasound, Fluoroscopy time, Dose-area product, Aortic aneurysm repair

## Abstract

**Objective:**

To assess the effect of using intravascular ultrasound (IVUS) in endovascular aortic aneurysms repair (EVAR) on reduction of radiation exposure.

**Methods:**

All EVAR procedures performed for symptomatic and asymptomatic infrarenal abdominal aortic aneurysms at a single U.K. center between January 2024 and December 2025 were retrospectively analyzed. Patients were divided into two groups based on imaging guidance used: IVUS-guided EVAR and standard EVAR. The primary end points were radiation dose and fluoroscopy time (FT), whereas the secondary outcomes included intraoperative technical success, change in pre- and postoperative serum creatinine level length of hospital stay, and 30-day morbidity and mortality.

**Results:**

During the study period, 51 patients met inclusion criteria at our center, 30 patients (58.8%) had standard EVAR procedure with fluoroscopy-guidance, whereas 21 patients (41.2%) had IVUS-guided EVAR procedure. Median dose area product in standard EVAR cases was 45.24 Gy cm^2^ compared with 7.27 Gy cm^2^ in IVUS-guided EVAR cases (*P* <.001, highly significant), and median FT in standard EVAR cases was 15.4 minutes compared with 4.5 minutes in IVUS-guided EVAR cases (*P* <.001, highly significant). Intraoperative technical success achieved in 19 patients (90%) in IVUS-guided cases compared with 28 patients (93%) in standard EVAR cases. The mean postoperative creatinine level was 71.4 ± 16.7 μmol/L in patients who underwent IVUS-guided EVAR cases, compared with 86.0 ± 22.0 μmol/L in patients with standard EVAR.

**Conclusions:**

IVUS provides accurate and safe graft deployment while reducing the total radiation dose and FT. This significantly reduces the radiation risks to patients and staff while maintaining the highest patient care and procedure safety.

**Article Highlights**

• **Type of Research:** Original research article, retrospective cohort study
• **Key Findings:** Intravascular ultrasound (IVUS) provides accurate and safe graft deployment while reducing total radiation dose and fluoroscopy time . This significantly reduces the radiation-related risks to patients and staff while maintaining the highest patient care and procedural safety.
• **Take Home Message:** In the current dominating endovascular era of aortic interventions, radiation safety becomes a great concern. IVUS-guided endovascular aneurysm repair was associated with lower radiation exposure than standard fluoroscopy-guided EVAR. The median dose-area product was 45.24 Gy cm^2^ in standard EVAR cases, compared with 7.27 Gy cm^2^ in IVUS-guided EVAR cases (*P* <.001, highly significant), and median fluoroscopy time in standard EVAR cases was 15.4 minutes compared with 4.5 minutes in IVUS-guided EVAR cases (*P* <.001, highly significant). These reductions may help minimize occupational radiation exposure among healthcare professionals. Furthermore, IVUS-guided EVAR was associated with improved kidney function, potentially owing to reduced contrast agent usage. This benefit may also be attributed to appropriate preoperative and postoperative intravenous fluid administration, adequate perioperative care, and optimal hydration management throughout the procedure.

Endovascular aortic aneurysms repair (EVAR) has become increasingly adopted as a minimally invasive treatment for infrarenal abdominal aortic aneurysms (AAAs), given its lower short-term morbidity and mortality and reduced length of hospital stay when compared with open surgical repair, but these procedures inevitably expose patients and operators to high-radiation doses, which cause cellular and tissue damages.^1^

International Commission on Radiological Protection and European Society for Vascular Surgery guidelines established the principles of radiation safety practice in endovascular operating rooms through the “As Low As Reasonably Achievable” (ALARA) concept. Ionizing radiation benefits during endovascular procedures should be weighed against the associated stochastic and deterministic risks of health effects by applying three principles: justification, optimization, and dose limits.^2^ Radiation safety could be achieved with developing the appropriate habits involving radiation use and protection through application of ALARA concept, which basically included three major concepts; first reduction of the number of images produced achieved by using pulse mode fluoroscopy, limitation of fluoroscopy time (FT) and addition of advanced imaging techniques (eg, image fusion), second reduction of radiation dose achieved by collimation, limitation of C-arm unit angulation and digital subtraction angiography (DSA) runs, avoidance of magnification or use digital magnification and accurate preprocedural planning; and third concept avoidance of unnecessary exposure achieved by usage of long sheaths to maximize operator distance from radiation source and strict application of shielding and protective garments. Usage of new technologies and modalities, which meet the ALARA concepts, such as intravascular ultrasound (IVUS) can be considered.^3^, ^4^

Radiation exposure-associated risks could be classified into deterministic and stochastic effects. Deterministic effects were considered as tissue reactions that could be observed when radiation dose thresholds were exceeded, despite being rare with low incidence after EVAR procedures, transient skin erythema at the X ray entry point can occur within 48 hours of the procedure when radiation skin exposure exceeds 2 Gy, whereas skin necrosis can be observed with skin doses more than 12 Gy several weeks after the procedure.^5^ Stochastic effects of radiation usually less predictable and associated with increase in long-term risks with either low or high doses, causing majorly cancer, the 15-year follow-up study of U.K. randomized controlled EVAR 1 trial showed significant increase in cancer mortality in the EVAR cohort.^6^

National U.K. survey of radiation doses during EVAR procedures recorded data from 3712 endovascular aortic procedures, including 2062 cases as standard EVAR, with median dose area product (DAP) of 162 Gy cm^2^.^7^ Usage of C-arm fluoroscopy guided unit in EVAR raised the issue of patient, surgeons, and working staff exposure to potentially high-radiation dose, which causing further morbidities and mortalities, as well as using large amount of contrast could degrade patients' kidney function and might end with dialysis, thus alternative ways with lower radiation exposure was adopted as IVUS-guided technique.^8^

The national consensus agreed that both DAP and FT are considered as the dose measurement tools to guide centers in evaluating their radiation safety. In this context, recent registered radiation dose as per national U.K. survey of radiation during EVAR was 162 Gy cm^2^, which considered as significantly high dose, which might jeopardize the procedure safety.^9^

Reduction in radiation exposure during EVAR should be a key priority for vascular surgeons. Awareness of the potential impact of radiation on both patients and the entire operating team is essential, thus alternative imaging modalities such as IVUS should be more widely adopted to minimize radiation exposure and enhance procedural safety.^10^ IVUS had been used since 1990s, providing real-time image during various aortic interventions including aneurysms, dissection, and penetrating aortic ulcers, it could confirm luminal passage and access to vessels with precise location of important branches and accurate vessels diameter measurements for successful graft deployment during EVAR, as well as identification of renal arteries and iliac bifurcation without C-arm unit angulations thereby reducing the radiation dose exposure and FT.^11^

This study aims to assess the radiation safety improvement associated with IVUS-guided EVAR compared with the standard fluoroscopy-guided EVAR.

## Methods

All patients who underwent EVAR procedure for infrarenal AAA in our center between January 2024 and December 2025 were retrospectively recruited for data collection. We included all asymptomatic infrarenal AAAs reaching threshold of intervention with diameter of ≥5.5 cm, as well as emergency symptomatic infrarenal AAA. We excluded all rupture infrarenal AAA, thoracic aortic aneurysms, and juxtarenal AAAs treated with thoracic EVAR (TEVAR) and fenestrated EVAR, respectively. These inclusion and exclusion criteria were based on the selection of standard infrarenal EVAR cases, which are not associated with more complexity than fenestrated EVAR or TEVAR cases. We also decided to compare the standard fluoroscopy-guided with IVUS based on literature review and recent guidelines, which appreciated the value of using IVUS to get precise location of renal arteries and internal iliac without need of angulation or large dose of contrast, also can be used to measure diameters and lengths of required grafts without using more fluoroscopy, which supports our concept in this study.

The collected study sample comprised patients who met our inclusion criteria and were classified into two groups. Group A included patients who underwent standard fluoroscopy-guided EVAR procedure, whereas group B included those patients who underwent IVUS-guided EVAR procedure, in which their endovascular database, theater logs, DAP (in Gy.cm^2^) as reflection of radiation doses, FT, duration of the procedure, and dose of contrast used, preoperative and postoperative creatinine level, 30-days mortality and morbidity as well as first 6-weeks postprocedural follow up CTA were extracted and analyzed.

The primary end points were radiation dose and FT, whereas the secondary outcomes included intraoperative technical success, which is defined as intraoperative complete exclusion of the aneurysm with no type I or III endoleak, change in pre- and postoperative serum creatinine level in micromole in liter, length of hospital stay, and 30-day morbidity and mortality. All patients followed up at our vascular outpatient clinic on regular intervals with follow-up scans.

In IVUS-guided group, we used a VOLCANO-Visions PV 0.035 IVUS catheter (Philips) in all cases to identify and mark the lowest renal artery and internal iliac arteries. The vessel diameter, characteristics, and device leg length were revised and confirmed compared with the previously decided measurements based on preoperative CTA, and final intraoperative DSA was performed using small amount of contrast to confirm the intraoperative technical success and exclusion of endoleak. In the standard C-arm-guided group, GE OEC Elite 31 Ergo Vascular C-arm(GE HealthCare) was used in majority of cases, and fewer cases done in Cath Lab using Philips Azurion Biplane system (Philips), where the lowest renal and both internal iliac were identified and marked using pump-contrast injection and fluoroscopy guided with significant angulations of craniocaudal, left-oblique angulations and right oblique angulations and magnifications, pre- and post-DSA were performed using 20 mL in 20 seconds using pump injector to exclude endoleak and intraoperative success.

In collaboration with our Department of Physics and Radiation Safety, radiation data and FT exposures were accurately collected, analyzed, and presented in our joint radiation governance meeting confirming the data accuracy and reliability. The data provided in the research are available in the result section, and for open-access usage, the research has been approved by the Research and Ethical Committee in our trust with collaboration with the Department of Vascular Surgery at the University Hospital of Coventry and Warwick, and it had been as well discussed in our quality improvement projects meeting and been approved. All patients had been consented and agreed for data publication in research project.

Data were collected, revised, coded, and entered the Statistical Package for Social Science (SPSS) version 27, (IBM Corp., released in 2020; IBM SPSS Statistics for Windows). The quantitative data were presented as means, standard deviations, and ranges for parametric data, and median and interquartile range when data found nonparametric. Also, qualitative variables were presented as number and percentages. The one-sample Kolmogorov-Smirnov test can be used to test that a variable is normally distributed. The comparison between groups regarding qualitative data was done by using *χ*^2^ *test* and/or *Fisher exact test* when the expected count in any cell found less than 5. The comparisons between two independent groups with quantitative data were performed using independent *t*-test for parametric distribution, and the Mann-Whitney *U* test for nonparametric distribution. The confidence interval was set to 95%, whereas *P* value <.05 was considered statistically significant.

The study aimed to evaluate the impact of IVUS-guided EVAR on radiation exposure during EVAR.

## Results

Out of the included 51 patients, 30 patients (58.8%) underwent standard fluoroscopy-guided EVAR, of whom 25 patients (83.3%) were male, with a mean age of 79.8 ± 6.6 years, whereas 21 patients (41.2%) underwent IVUS-guided EVAR procedure, 13 of them (61.9%) were male with a mean age of 79.6 ± 6.8 years (Table I).

**Table I:** Comparison between the intravascular ultrasound (*IVUS*)-guided endovascular aortic aneurysms repair (*EVAR*) group and standard EVAR group regarding demographic data

	IVUS-guided EVAR	Standard EVAR	Test value	*P* value	Significance
	N = 21	N = 30			
Age, years			−0.113^a^	.911	NS
Means ± SD	79.62 ± 6.77	79.83 ± 6.63			
Range	64-93	66-90			
Sex			2.987^b^	.084	NS
Male	13 (61.9)	25 (83.3)			
Female	8 (38.1)	5 (16.7)			

*SD*, Standard deviation.

*P* > .05, Nonsignificant (NS); *P* < .05, significant; *P* < .01, highly significant.

Categorical data are shown as number (%).

a Independent *t*-test.

b *χ*^2^ test.

In the standard EVAR group, the mean aortic aneurysm diameter was 5.8 ± 1.0 cm. A GORE graft was used in 16 patients (53.3%), a Medtronic graft in eight patients (26.7%), an ALTO graft in three patients (10.0%), and a COOK device in two patients (6.7%). In the IVUS-guided group, the mean aortic aneurysm diameter was 5.8 ± 1.0 cm. Majority of the cases treated using GORE graft (16 patients, 76.2%), whereas Medtronic graft was used in five patients (23.8%; Table II).

**Table II:** Comparison between the intravascular ultrasound (*IVUS*)-guided endovascular aortic aneurysms repair (*EVAR*) group and standard EVAR group regarding operative characteristics

	IVUS-guided EVAR	Standard EVAR	Test value	*P* value	Significance
	N = 21	N = 30			
AAA diameter, cm			−1.487^a^	.143	NS
Means ± SD	5.78 ± 1.02	6.16 ± 0.81			
Range	4-8.5	4.5-9			
Graft used			5.268^b^	.261	NS
GORE	16 (76.2)	16 (53.3)			
Medtronic	5 (23.8)	8 (26.7)			
ALTO	–	3 (10.0)			
COOK	–	2 (6.7)			
Not available	–	1 (3.3)			
Graft size			−1.896^a^	.064	NS
Means ± SD	26.1 ± 3.26	27.67 ± 2.62			
Range	20-32	23-36			

*AAA*, Abdominal aortic aneurysms; *SD*, standard deviation.

*P* > .05, nonsignificant (NS); *P* < .05, significant; *P* < .01, highly significant.

Categorical data are shown as number (%).

a Independent *t* test.

b Fisher exact test.

When we analyzed the radiation exposure data extracted from the C-arm X ray unit during both types of procedures, we found that in standard EVAR group, median DAP was 45.24 Gycm^2^ and FT was 15.4 minutes, compared with DAP 7.27 Gy cm^2^ and FT 4.5 minutes in the IVUS-guided group (*P* value highly significant <.001; Table III).

**Table III:** Comparison between the intravascular ultrasound (*IVUS*)-guided endovascular aortic aneurysms repair (*EVAR*) group and standard EVAR group regarding dose area product (*DAP*; Gy cm^2^) and fluoroscopy time (*FT*; minutes)

	IVUS-guided EVAR	Standard EVAR	Test value	*P* value	Significance
	N = 21	N = 30			
FT, minutes			−3.855^a^	<.001	HS
Median [IQR]	4.52 [3.40-8.43]	15.38 [8.22-20.09]			
Range	1.56-19.45	4.09-68.19			
DAP, Gy cm^2^			−5.627^a^	<.001	HS
Median [IQR]	7.27 [5.63-10.07]	45.24 [23.29-61.91]			
Range	3.16-27.43	12.09-166.54			

*IQR*, Interquartile range.

*P* > .05, Nonsignificant; *P* < .05, significant; *P* < .01, highly significant (HS).

a Mann-Whitney *U* test.

Our results showed that IVUS-guided EVAR procedure was associated with lower radiation exposure to the patients when compared with standard fluoroscopy-guided EVAR (Figs 1 and 2). This will result in reduced environmental radiation exposure to staff.

**Fig 1:**
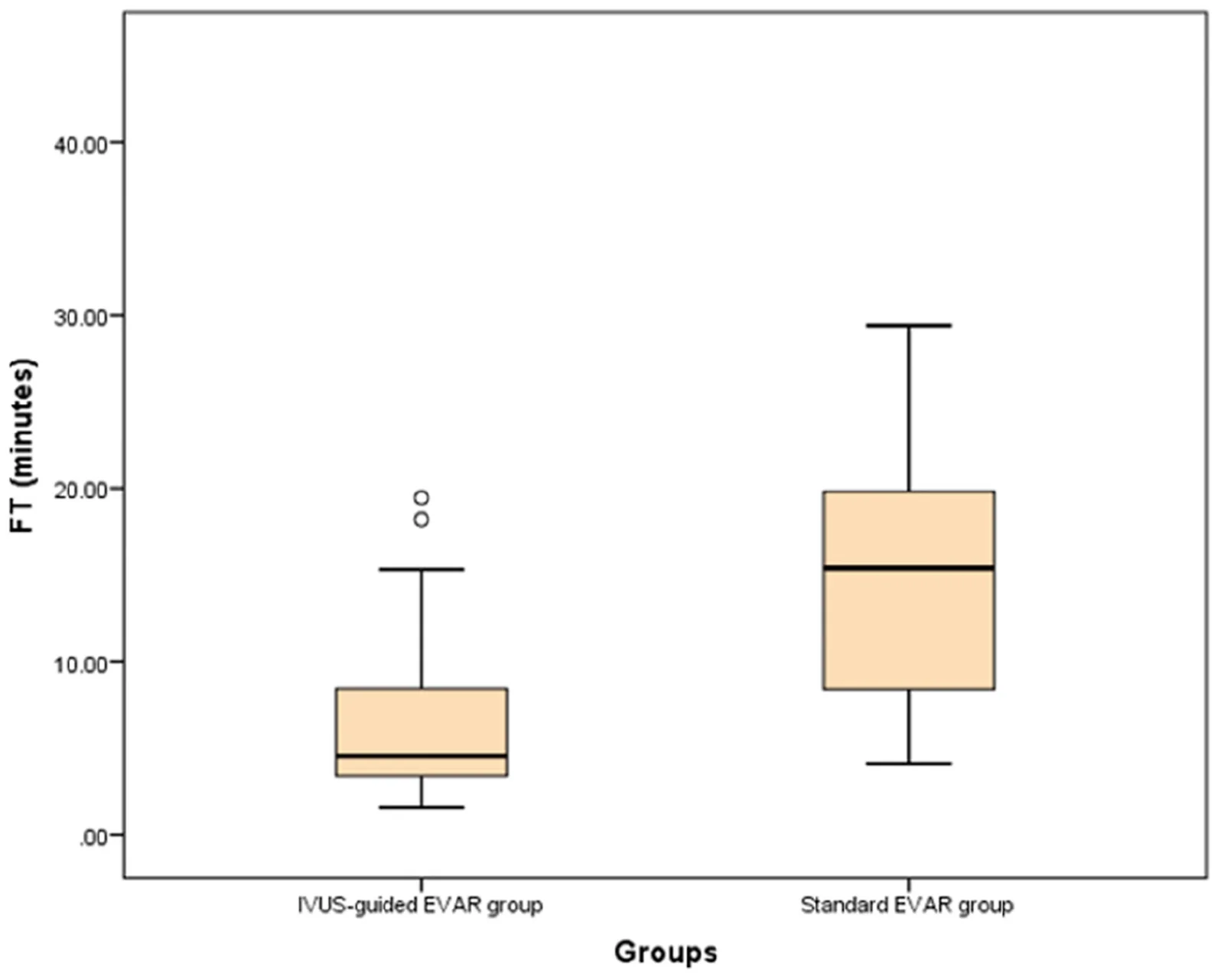
Comparison between the intravascular ultrasound (*IVUS*)-guided endovascular aortic aneurysms repair (*EVAR*) group and standard EVAR group regarding fluoroscopy time (*FT*; minutes) using interquartile range (IQR).

**Fig 2:**
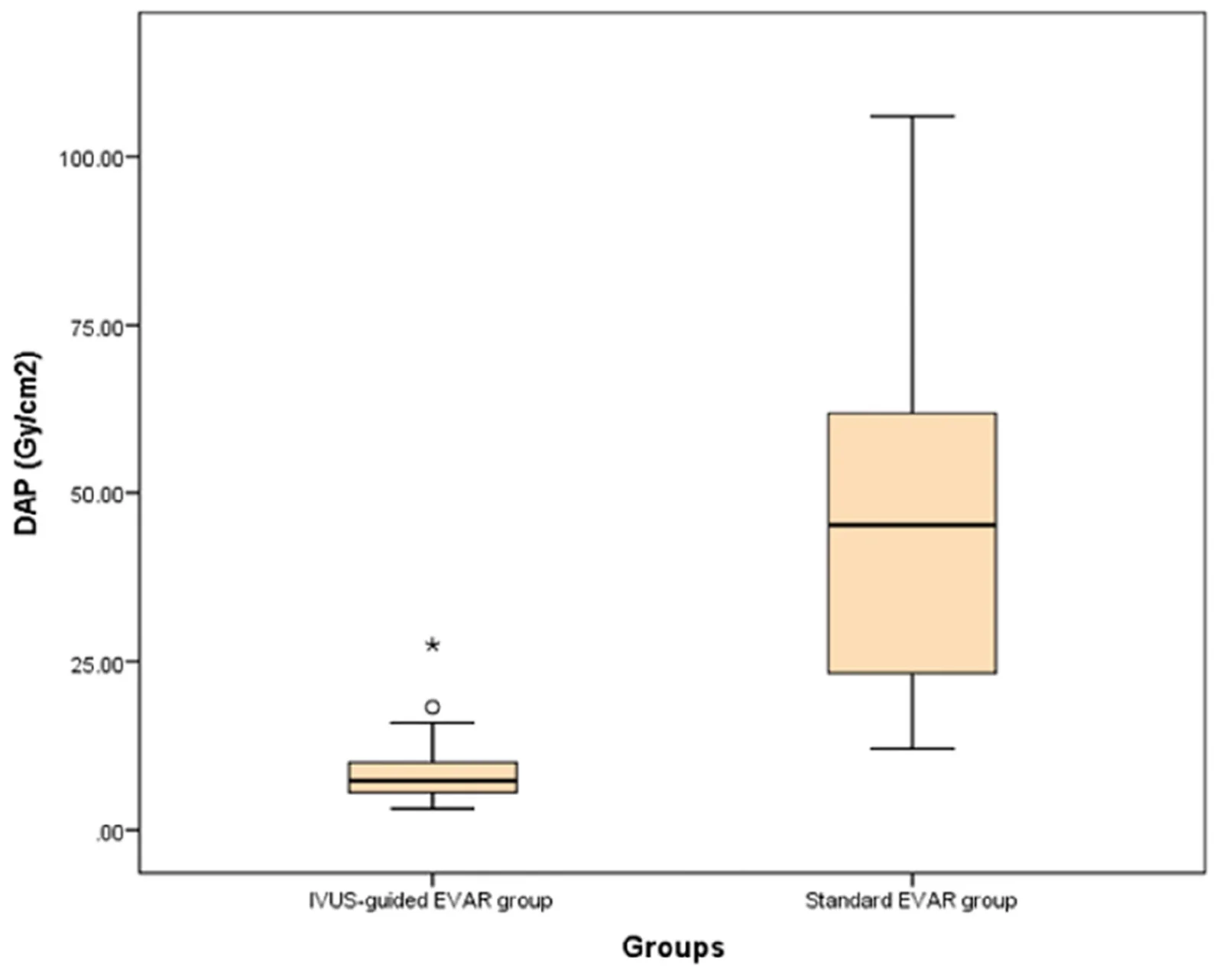
Comparison between the intravascular ultrasound (*IVUS*)-guided endovascular aortic aneurysms repair (*EVAR*) group and standard EVAR group regarding dose area product (*DAP*; Gy cm^2^) using interquartile range (IQR).

We found that median contrast dose used in standard EVAR group was 140 mL of Visipaque 270 compared with 50 mL in IVUS-guided group, and that could be reflected on the change in pre and postoperative creatinine level measured in micromoles per liter (μmol/L). In standard EVAR group, postoperative creatinine showed mild improvement compared with preoperative −6.0% (−13.2 to −3.5), whereas in IVUS-guided group, postoperative creatinine showed significant improvement with reduction percent −15 (−19.63 to −7.04; Table IV; Fig 3). Our data showed improvement of kidney function in both groups, owing this to usage low dose of contrast, as well as proper preoperative and postoperative intravenous fluid replacement and proper perioperative care with good optimization of patients' hydration through the whole process. Fig 4 showed significant improvement in postoperative creatinine level compared with preoperative levels in IVUS-guided EVAR group, whereas no significant improvement in preoperative and postoperative creatinine level in standard fluoroscopy-guided EVAR group as well as no significant difference between the two groups, yet this perioperative creatinine level improvement in IVUS-guided group could not be definitively owed solely to low-contrast dose used but also because of other factors impacting, such as intraoperative fluids, patient preoperative hydration status, and perioperative hemoglobin levels.

**Table IV:** Comparison between the intravascular ultrasound (*IVUS*)-guided endovascular aortic aneurysms repair (*EVAR*) group and standard EVAR group regarding preoperative and postoperative creatinine levels

	IVUS-guided EVAR	Standard EVAR	Test value	*P* value	Significance
	N = 21	N = 30			
Preoperative creatinine			−0.760^a^	.451	NS
Means ± SD	85.48 ± 25.14	90.57 ± 22.37			
Range	48-138	57-144			
Postoperative creatinine			−2.550^a^	.014	S
Means ± SD	71.43 ± 16.66	86.0 ± 21.99			
Range	44-107	55-131			
Paired *t-*test					
Test value	3.448	1.222			
*P* value	0.003	0.232			
Percentage of reduction			2.431	.015	S
Means ± SD	−15 (−19.63 to −7.04)	−5.95 (−13.21 to −3.51)			
Range	−55.04 to 51.79	−100.0 to 75.76			

*SD*, Standard deviation.

*P* > .05, Nonsignificant (NS); *P* < .05, significant (S); *P* < .01, highly significant.

a Independent *t*-test.

**Fig 3:**
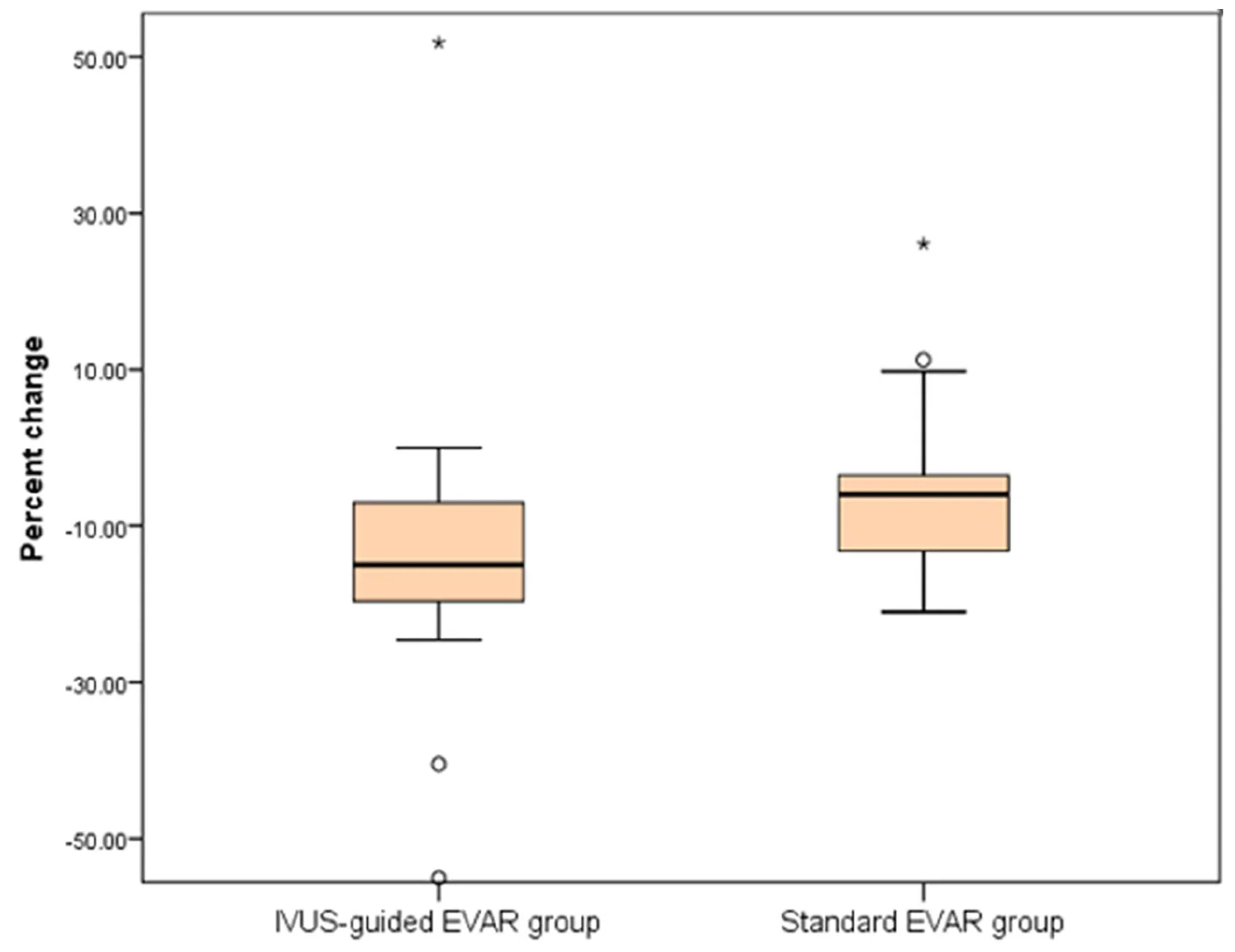
Comparison between the intravascular ultrasound (*IVUS*)-guided endovascular aortic aneurysms repair (*EVAR*) group and standard EVAR group regarding percent change of creatinine level using interquartile range (IQR).

**Fig 4:**
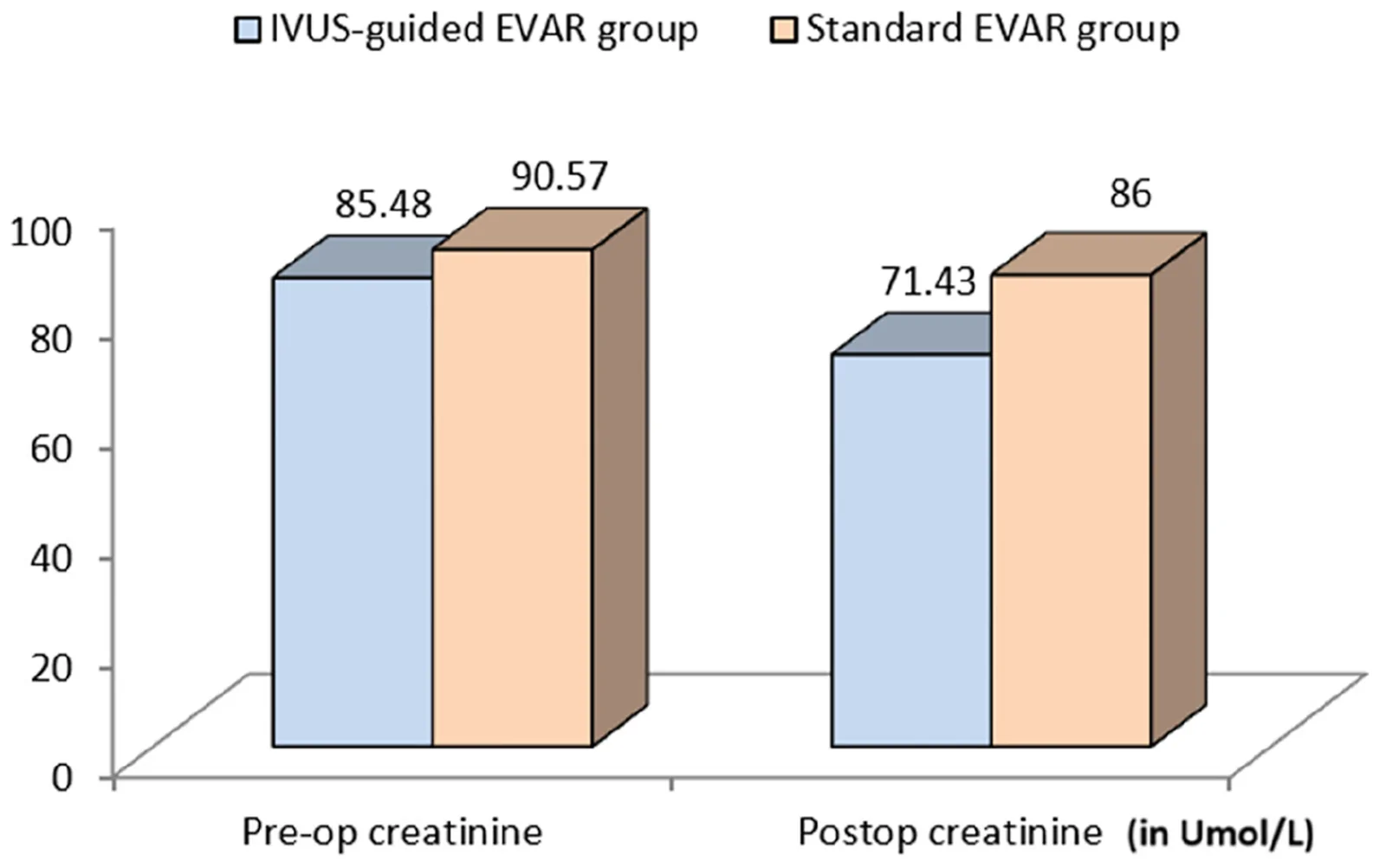
Comparison between the intravascular ultrasound (*IVUS*)-guided endovascular aortic aneurysms repair (*EVAR*) group and standard EVAR group regarding pre and postoperative creatinine (in μmol/L).

Intraoperative technical success achieved in 19 patients (90%) in IVUS-guided group, as two patients (9.5%) on final angiogram showed type IA endoleak and aortic cuff was added with proper sealing on final angiogram and complete exclusion of the aneurysm, whereas two patients developed type II endoleaks, which were conservatively treated. In the standard EVAR group, technical success was achieved in 28 patients (93%). Two patients (6.6%) developed type IA endoleaks, which were successfully sealed with aortic cuff. Three patients (10%) showed type 1A endoleak that were managed with prolonged aortic ballooning, without the need for an aortic cuff or graft extension. The median procedure duration was 2.5 hours in the standard EVAR group and 2 hours in the IVUS-guided EVAR group. However, taking into consideration that these durations included the total procedure time, not only the EVAR, which might associate in some cases with femorofemoral crossover bypass, or common femoral artery endarterectomy with patch repair.

The median length of hospital stay was 3 days in both groups. In the IVUS-guided EVAR group, 30-day morbidity was 4.8%, and no 30-day mortality occurred. In the standard EVAR group, 30-day morbidity was 6.7%, and 30-day mortality was 3.3%, as one patient died with acute myocardial infarction showing no statistically significant difference between the two groups. During follow-up as per protocol, data were collected from postprocedure 6-week CTA. In the IVUS-guided group, seven patients (33.3%) developed type II endoleaks with no sac expansion and no reintervention needed, whereas in the standard EVAR group, nine patients (30%) developed endoleaks, seven patients had type II endoleaks with no sac expansion and no reintervention needed. One patient developed type I endoleak and underwent aortic cuff insertion, and one patient developed iliac limb stenosis and underwent iliac angioplasty.

## Discussion

Various studies have shown the effect of IVUS on radiation dose reduction and procedural safety improvement, in some cases with zero-contrast usage. Over the past decade, IVUS has proved efficiency in EVAR procedures and aortic dissection cases. Its advantages over angiographic guidance techniques have became apparent, as IVUS does not require oblique projections or angulations, thereby reducing the radiation dose without differences in procedural outcome.^12^ IVUS is considered as an efficient tool in the assessment of both distal and proximal stent graft-landing zones, as it provides 360° visualization of the arterial wall, distinguishes the plaque nature, and precisely locates anatomical arteries and branches without the need of angulations associated with higher dose of radiation. Also, it gives data in real-time view that helps in final graft selection through reliable intraluminal diameter and length measurements, enabling accurate graft deployment and potentially decreasing the incidence of endoleaks.^13^ The frequent use of IVUS with increasing rate of paper publishments showed that this technique has been more efficient than the standard fluoroscopy-guided EVAR within identification of the ostia of iliac and renal arteries as well as removing the problem of parallax in an anteroposterior view of angiography that are associated with lower radiation doses and FT and increase the procedure safety for both the patient and the medical team.^14^

The *Journal of Vascular Surgery* in association with the Society for Vascular Surgery recently published a paper regarding the efficacy of using IVUS in EVAR procedures with respect to low-contrast dose usage and lower radiation exposure; 90 patients were recruited from January 2023 to August 2024, the results showed the mean radiation dose was 525 ± 291 mGy cm^2^ in the IVUS-guided EVAR compared with 827 ± 496 mGy cm^2^ in the standard EVAR (*P* = .0007), which were significantly reduced with IVUS.^15^

Over the past decade, IVUS progressed from being a diagnostic tool in peripheral arterial disease cases or aortic dissection cases to an efficient guiding and therapeutic tool for EVAR procedures.^16^ Despite being not commonly used because of lack of intraoperative endoleaks detection, the study published in Italy in 2022 showed its IVUS value in EVAR procedures with regard to radiation safety and low-contrast dose usage. This study included 35 patients with infrarenal AAA who underwent IVUS-guided EVAR and compared them with 35 consecutive patients who underwent standard EVAR. All patients achieved a minimum follow-up of 12 months. The results showed that mean radiation dose was 700 mGy cm^2^ in the IVUS-guided EVAR group, compared with 1200 mGy cm^2^ in the standard EVAR group. The mean FT was 18.5 minutes in the IVUS-guided group compared with 30 minutes in the standard EVAR group, with a statistically significant difference (*P* < .05). No contrast medium was used in the IVUS-guided group compared with a mean of 125 mL in the control group.^17^

Recently published study regarding the utilization of IVUS in EVAR and TEVARs in the United States during a period of time between 2016 and 2023, a total of 136,540 patients underwent TEVAR and EVAR, of which 9.8% patients (13,364) used IVUS, which was associated with lower risk of radiation exposure, and lower rate of reintervention, also there was a subgroup analysis suggested lower risks of malperfusion, thoracoabdominal dissection with malperfusion, and chronic kidney disease development in the IVUS-guide group.^18^

Although more studies and clear guidelines are still required to validate the IVUS usage in the EVAR procedures, but by comparing recent published papers with our study results, we showed that IVUS could be used efficiently and safely EVAR procedures, also we showed that radiation exposure and FT are lower than most of national and international values.

Our study limitation is being single-center experience, so our results cannot be generalized, but we covered acceptable time duration with acceptable study sample. When our trust suggested using IVUS during EVAR as a new institutional change of practice to minimize radiation exposure guided by recent guidelines and literature review, a limitation encountered was surgeons experiences and familiarization to ultrasound anatomical landmarks. This was managed by attending workshops liaised and led by Philips company, and cases were undertaken by two consultants until sufficient experience was gained.

## Conclusions

IVUS provides accurate and safe graft deployment while reducing the total radiation dose and FT. This significantly reduces the radiation risks to patients and staff while maintaining the highest patient care and procedure safety.

## Author Contributions

Conception and design: MS, AhE, AM, NM, AH, CK, AsE

Analysis and interpretation: MS, AM, NM, CK, AsE

Data collection: MS, AhE, CK, AsE

Writing the article: MS, AsE

Critical revision of the article: MS, AhE, AM, NM, AH, CK, AsE

Final approval of the article: MS, AhE, AM, NM, AH, CK, AsE

Statistical analysis: MS

Obtained funding: Not applicable

Overall responsibility: MS.

## Ethical approval

The study was approved by the Research and Ethical Committee of the Department of Vascular Surgery at the University Hospital of Coventry and Warwick.

## Consent for publication

All patients provided consent and agreed for data publication.

## Data availability

All data are available in the research and for open-access usage.

## Funding

None.

## Disclosures

None.

## References

[1] Modarai B., Haulon S., Ainsbury E. (2023). Editor's choice–European Society for Vascular Surgery (ESVS) 2023 clinical practice guidelines on radiation safety. Eur J Vasc Endovasc Surg.

[2] Sharma A., Sethi P., Gupta K. (2020). Endovascular abdominal aortic aneurysm repair. Interv Cardiol Clin.

[3] Mastracci T.M. (2023). The evolved surgeon: ESVS radiation guidelines mark the next era in vascular care. Eur J Vasc Endovasc Surg.

[4] (2007). Annals of the ICRP, publication 103, the 2007 recommendations of the International Commission on Radiological Protection, Volume 37 Nos 2-4 2007, ISSN 0146-6453, ISBN 978-0-7020-3048-2. CRP, 2007. ICRP Publication 103. Ann ICRP.

[5] ICRP (2012). ICRP statement on tissue reactions/early and late effects of radiation in normal tissues and organs–threshold doses for tissue reactions in a radiation protection context. ICRP publication 118. Ann ICRP.

[6] The United Kingdom EVAR Trial Investigators (2010). Endovascular versus open repair of abdominal aortic aneurysm. N Engl J Med.

[7] Tsitsiou Y., Velan B., Ross R., Lakshminarayan R., Rogers A., Hamady M. (2024). National UK survey of radiation doses during endovascular aortic interventions. Cardiovasc Intervent Radiol.

[8] Rial R., Vañó E., Río-Solá M.L.D. (2020). National diagnostic reference levels for endovascular aneurysm repair and optimisation strategies. Eur J Vasc Endovasc Surg.

[9] Hertault A., Rhee R., Antoniou G.A. (2018). Radiation dose reduction during EVAR: results from a prospective multicentre study (The REVAR study). Eur J Vasc Endovasc Surg.

[10] Pecoraro F., Bracale U.M., Farina A. (2019). Single-center experience and preliminary results of intravascular ultrasound in endovascular aneurysm repair. Ann Vasc Surg.

[11] Foerth M., Seidenbusch M.C., Sadeghi-Azandaryani M., Lechel U., Treitl K.M., Treitl M. (2015). Typical exposure parameters, organ doses and effective doses for endovascular aortic aneurysm repair: comparison of Monte Carlo simulations and direct measurements with an anthropomorphic phantom. Eur Radiol.

[12] Peng C., Wu H., Kim S., Dai X., Jiang X. (2021). Recent advances in transducers for intravascular ultrasound (IVUS) imaging. Sensors.

[13] Taalab M.A., Kamal A.M., Mohammad A.F., Zaki M.M. (2024). Intravascular ultrasound versus computed tomography angiography in sizing and operative management of endovascular aortic aneurysm repair. J Endovasc Ther.

[14] Kobak J.E., Wallach W., Oskroba A. (2024). Intravascular ultrasound (IVUS) in endovascular abdominal aortic aneurysm repair - an adjunctive imaging tool or even less invasive self-guidance method?. Acta Angiol.

[15] Oga Y., Kuratani T., Kudo T. (2025). No More Contrast Medium! The Efficacy of IVUS-Guided EVAR. J Vasc Surg.

[16] Karaolanis G.I., Antonopoulos C.N., Georgakarakos E. (2022). Colour duplex and/or contrast-enhanced ultrasound compared with computed tomography angiography for endoleak detection after endovascular abdominal aortic aneurysm repair: a systematic review and meta-analysis. J Clin Med.

[17] Fanelli F., Cannavale A., Falcone G., Citone M., Annese A., O’ Sullivan G. (2022). Evar under IVUS guidance: zero contrast and low radiation dose. LOJ Med Sci.

[18] Mosarla R.C., Heindel P.V., Hussain M.A. (2025). Utilization and outcomes associated with intravascular ultrasound during abdominal and thoracic endovascular aortic interventions in the United States in the Contemporary era (2016-2023). Circ Cardiovasc Interv.

